# The effects and mechanism of environmental enrichment on MK-801 induced cognitive impairment in rodents with schizophrenia

**DOI:** 10.3389/fncel.2022.1024649

**Published:** 2022-09-29

**Authors:** Jinwei Xu, Yaohao Li, Biqing Tian, Haiying Liu, Shengxi Wu, Wenting Wang

**Affiliations:** Department of Neurobiology, School of Basic Medicine, Air Force Medical University, Xi'an, China

**Keywords:** schizophrenia, environmental enrichment, MK-801, cognitive symptoms, mechanism

## Abstract

Schizophrenia is a severe mental disorder characterized by positive, negative, and cognitive symptoms. Cognitive symptoms are a kind of symptoms with high incidence and great impact on patients. There is no effective treatment in clinical practice. N-methyl-d-aspartic acid (NMDA) receptor hypofunction may be an important cause of cognitive symptoms. MK-801 (also named Dizocilpine), a noncompetitive antagonist of NMDA receptor, is often used to construct a model of NMDA receptor dysfunction. In terms of treatment, environmental enrichment (EE) as an environmental intervention can effectively improve the symptoms of cognitive impairment in rodents. In this paper, we first briefly introduce the background of cognitive symptoms and EE in schizophrenia, and then investigate the manifestations of MK-801 induced cognitive impairment, the improvement of EE on these cognitive impairments based on the MK-801 induced schizophrenia rodent models, and the possible mechanism of EE in improving cognitive symptoms. This article reviews the literature in recent years, which provides an important reference for MK-801 to construct a cognitive symptom model of schizophrenia and the mechanism of EE in improving cognitive symptoms of schizophrenia.

## Introduction

Schizophrenia (SCZ) is a severe mental disease characterized by positive symptoms (delusions, hallucinations), negative symptoms (anhedonia, social withdrawal), and cognitive symptoms (learning, and memory disorders), which affects about 1% of the world's population. It is one of the most serious mental disorders (Jauhar et al., 2022). Cognitive symptoms are the core symptoms of schizophrenia, which have a great impact on the quality of life of patients, with a high incidence in patients, and up to 98.1% of patients have cognitive disorders (Wu et al., 2021). Moreover, there is a lack of effective treatment for cognitive symptoms. Clinically, anti-schizophrenic drugs are effective for positive symptoms, but the efficacy of drugs will reduce when treating negative symptoms. However, the therapeutic effect of these drugs on cognitive symptoms is negligible (Prades et al., 2017). Due to the great impact on patients and the lack of effective treatment, cognitive symptoms have attracted more and more attention in the latest research on schizophrenia. The pathological mechanism of schizophrenia is complex. Since the 1980s, clinical studies and animal model studies have suggested that schizophrenia is closely related to the impairment of glutamate transmission in the brain. Therefore, the glutamate hypothesis has been proposed, which suggests that NMDA receptor may be the key molecules of schizophrenia (Hansen et al., 2018). Decreased mRNA expression levels of NMDA receptor and abnormal receptor binding sites can cause glutamate dysfunction, resulting in cognitive deficits (Li et al., 2018). Clinical studies have found that NMDA receptor antagonists in healthy individuals can induce cognitive impairment symptoms similar to those of schizophrenia patients and can aggravate the original symptoms of schizophrenia (Adell, 2020). Laboratory animal model studies have also shown that NMDA receptor antagonists can induce cognitive impairment symptoms in animal models, which have been widely used in the study of schizophrenia (Vales and Holubova, 2021).

Building animal models is the most common way to study schizophrenia. Based on the inducement of schizophrenia, common animal models of schizophrenia include developmental models, genetic models, and drug models (Winship et al., 2019). Among them, the drug models are the most common animal models and non-competitive antagonists of NMDA receptor are commonly used to construct drug models (Winship et al., 2019). MK-801 is a non-competitive antagonist of the NMDA receptor. Compared with other commonly used non-competitive antagonists of NMDA receptor, such as phencyclidine and ketamine, MK-801 has a stronger inhibitory effect on NMDA receptor (Wallach et al., 2016), with higher affinity and specificity (Nakazawa and Sapkota, 2020). In addition, MK-801 can simulate the typical positive symptoms, negative symptoms, and cognitive symptoms of schizophrenia in animals at the same time, which is considered to be one of the best drugs for schizophrenia modeling (Shahzad et al., 2017).

Environmental enrichment (EE) is an environmental intervention that can limit the presentation of schizophrenia symptoms in a rat model of the disorder (Zhu and Grace, 2021). Due to the obvious limitations of drug treatment, non-drug means such as cognitive behavioral therapy (Batinic, 2019) and exercise therapy (Girdler et al., 2019) to control schizophrenia are gradually being paid attention to. EE can provide rodents with an environment that promotes physical activity, cognitive activity, and social interaction, which can have beneficial effects on the structure and function of the brain, behavior, cognition, and other aspects (Livingston-Thomas et al., 2016; Gubert and Hannan, 2019). The key of EE is offering an innovative environment, social contact, and exercise facilities (Crofton et al., 2015). The basic experimental settings include feeding the rodents in the larger community and bigger cages, adding some toys in the cage, nesting material, pipes and other items used for the rats or mice climbing and hiding, and regular change of squirrel cage inside decorate (Kempermann, 2019). EE has been proven to improve behavioral abnormalities in rodent models of schizophrenia and improve complex cognitive functions such as learning and memory (Burrows et al., 2015).

This article will review the cognitive symptoms of MK-801 induced in rodents, the effect of EE on cognitive impairment behavior, and the possible mechanisms.

## MK-801 induced cognitive impairment in rodents with schizophrenia

Cognitive impairment is the core feature of schizophrenia, which seriously affects the quality of life of patients (Maas et al., 2020). NMDA receptor is a ligand-gated ionic glutamate receptor mainly present in the central nervous system, and NMDA receptor-mediated glutamate transmission is believed to be related to cognitive function, especially in learning and memory (Khakpoor et al., 2021). MK-801 is a noncompetitive antagonist of the NMDA receptor, which is considered to be a cognition impairer, a compound that disrupts cognitive function, causing learning, memory, and sensorimotor gating dysfunction (Rahati et al., 2016). These defective behaviors can be detected by the new object recognition (NOR) experiment, Morris water maze, object-in-place experiment, passive avoidance (PA) test, eight-arm radial maze, and prepulse inhibition (PPI). The results can be used to assess whether the cognitive function of rodents is normal.

There are a variety of classification methods for memory function, one of which can divide memory function into working memory and reference memory. Working memory includes short-term memory and spatial memory, etc., while reference memory is considered a long-term and permanent memory or habit (Bernaud et al., 2021). Different experiments detect the two kinds of memory abilities differently. NOR, Morris water maze and object-in-place experiment are used to detect working memory defects, PA test and eight-arm radial maze can detect not only working memory but also reference memory. In the following, the MK-801 induced cognitive deficit behaviors will be reviewed in order of experimental detection of different memories (see Table 1).

**Table 1:** MK-801 induced cognitive symptoms of schizophrenia in rodents and improvement of cognitive symptoms by EE.

**Experiment**	**Drug intervention**	**Sex/strain**	**Behavior change**	**EE intervention**	**Behavior improvement**	**Bibliography**
NOR	Chronic 0.1 mg/kg MK-801	Male and female C57BL/6 neonatal mice	Discrimination index declines			Plataki et al., 2021
	Acute 0.2 mg/kg MK-801	Male C57/BL6J mice	Discrimination index declines			Prades et al., 2017
	Chronic 0.5 mg/kg MK-801	Male and female Long Evans neonatal rats	Discrimination index declines	18 days EE treats during P55-P73	The discrimination index of the MK-801 treated group and control group increased	Murueta-Goyena et al., 2018
	Chronic 1 mg/kg MK-801	Male and female Wistar neonatal rats	Recognition index declines	EE treats on the first day after birth until the end of the experiment	The male behavior improves significantly, but the female does not improve	Faatehi et al., 2019
Morris water maze	Chronic 0.5 mg/kg MK-801	Male Long Evans neonatal rats	Increase in swimming distance, increase in time spent	EE treats on the first day after birth until the end of the experiment	Swimming distance and time spent decrease	Murueta-Goyena et al., 2019
	Chronic 1 mg/kg MK-801	Male Wistar neonatal rats	Increase in swimming distance, increase in time spent	EE treats on the first day after birth until the end of the experiment	Swimming distance and time spent decrease in the MK-801 treated group and control group	Nozari et al., 2014
	Chronic 0.25 mg/kg MK-801	Male Balb/c neonatal mice	Increase in swimming distance, increase in time spent	EE treats on the 21st day after birth until the end of the experiment	EE does not rescue the impaired performance of spatial learning ability	Akillioglu et al., 2012
Object- in-place	Chronic 0.5 mg/kg MK-801	Male Long Evans neonatal rats	Discrimination index declines	EE treats on the first day after birth until the end of the experiment	Discrimination index increases	Murueta-Goyena et al., 2019
PA test	Acute 0.15 mg/kg MK-801	Male Wistar rats	Long-term memory is impaired			Zahiri et al., 2021
	Chronic 0.25 mg/kg MK-801	Male Wistar neonatal rats	Both long-term and short-term memory are normal			Kocahan et al., 2013
	Chronic 1 mg/kg MK-801	Male Wistar neonatal rats	Long-term memory is impaired, short-term memory is normal	EE treats on the first day after birth until the end of the experiment	Incubation period increases and long-term memory improves	Rahati et al., 2016
Eight-arm radial maze	Chronic 0.5 mg/kg MK-801	Male Wistar rats	Working memory and reference memory are impaired			Li et al., 2019
	Acute 0.08 mg/kg MK-801	Male Long-Evans rats	Spatial working memory is impaired			Nishiyama et al., 2017
	Chronic 1 mg/kg MK-801	Female Wistar neonatal rats	Working memory is impaired, reference memory is normal	EE treats on the first day after birth until the end of the experiment	No improvement in impaired working memory	Nozari et al., 2015a
PPI	Acute 0.2 mg/kg MK-801	Male C57/BL6J mice	PPI decreases at 81, 85, 90 dB			Prades et al., 2017
	Chronic 1 mg/kg MK-801	Male and female Wistar neonatal rats	Male and female rats show PPI deficiency	EE treats on the first day after birth until the end of the experiment	PPI deficiency is improved in male rats, but not in female rats	Nozari et al., 2015b

NOR is an experiment that uses the rodents' stronger tendency to explore new objects to assess their working memory. If the animal has a good memory function, it will spend more time exploring new objects, whereas the animal with memory problems will spend more time on old objects. This experiment is mainly used to measure hippocampal function as well as the function of other cortical regions involved in object recognition, such as the prefrontal cortex (Denninger et al., 2018). Chronic treatment with 1 mg/kg MK-801 in the early stage of Wistar neonatal rats can reduce the recognition index in NOR, which shows that the time to explore the novel objects is decreased, while the time of exploring the old objects is increased. And the performance of male and female Wistar neonatal rats is similar (Faatehi et al., 2019). Treatment of C57BL/6 neonatal mice with low concentrations of MK-801, such as 0.1 mg/kg, has similar effects (Plataki et al., 2021). However, it has also been argued that chronic MK-801 administration induces working memory deficits in NOR with a threshold of administration concentration, and only concentrations higher than 0.5 mg/kg can successfully induce working memory deficits (Murueta-Goyena et al., 2018). In addition, the time of MK-801 administration also affects the NOR performance. For example, C57BL/6 neonatal mice show impaired discrimination index when MK-801 is administered on postnatal days 7–14, but there is no significant difference in NOR results between MK-801 treated and control mice when MK-801 is administered on postnatal days 11–15. This suggests that there is a time window for the effect of MK-801 on cognitive function (Plataki et al., 2021). In addition, acute MK-801 treatment also reduces the discrimination index of male C57/BL6J mice (Prades et al., 2017).

Morris water maze is an experiment to access learning and spatial memory by finding an escape route in the pool. Spatial memory is a type of working memory. If the learning and memory function is normal, the rodents will spend less time and swim less distance from being put into the water maze to boarding the platform after the they were trained for several days (Lissner et al., 2021). Compared with the control group, the Long Evans neonatal rats in the MK-801 treatment group take more time to find the hidden platform and have a longer swimming distance, indicating that their spatial learning ability is impaired (Murueta-Goyena et al., 2019). Treatment of adult C57BL/6 mice with MK-801 results in similar impaired spatial learning (Xiao et al., 2019).

Object-in-place experiment is an experiment to detect the spatial location memory of objects. The principle is that rodents will spontaneously remember the location of objects in space. When the original object's location moves, they will spend more time exploring the moved object (Aggleton and Nelson, 2020). The spatial location memory is also a kind of working memory. The discrimination index of Long Evans neonatal rats in the MK-801 treatment group is significantly reduced, and the object location memory is impaired (Murueta-Goyena et al., 2019).

The PA test is an experiment to evaluate working memory and reference memory function. The experimental device is composed of a light room and a dark room. During the training process, the rat or mouse will be put into the light room first, and a plantar shock will be given to them after they enter the dark room. Tests performed 2 h after training are used to assess short-term memory, whereas longer tests such as 24 h later are used to assess long-term memory. Short-term memory and long-term memory, respectively belong to working memory and reference memory (Kruk-Slomka and Biala, 2016). PA test only requires short-term training, which avoids memory overlap between experimental and training stages, but electrical stimulation can cause some harm to animals. The results of the PA test show that MK-801 can cause the impairment of PA memory and shorten the latency entering a dark room, and the response effect of the PA test to MK-801 is dose-dependent (van der Staay et al., 2011). Chronic treatment of Wistar neonatal rats with 1 mg/kg MK-801 mainly shows long-term memory impairment, with little effect on short-term memory (Rahati et al., 2016), but some studies also find that chronic treatment with 0.25 mg/kg MK-801 has no effect on the PA memory. There is no significant difference in short-term memory and long-term memory compared with the control group (Kocahan et al., 2013). Acute MK-801 administration results in a significantly shorter latency to enter the dark room when tested at 24 h, 48 h, and 10 d after training, indicating impairment of long-term memory in Wistar rats (Zahiri et al., 2021).

The eight-arm radial maze is an experiment used to assess working memory and reference memory (Mei et al., 2020). In experiments, rodents tend to explore the arm of the maze with food, and correct behavior means that they visit each arm of the maze with food only once (Kurzina et al., 2020), entering the arm without food indicates faulty reference memory, and re-entering the visited arm indicates faulty working memory (Kim et al., 2018). Chronic MK-801 treatment can damage both working memory and reference memory in male Wistar rats (Li et al., 2019), but chronic MK-801 treatment can only damage working memory and has no effect on reference memory in female Wistar rats. The reason for this situation may be that estrogen in the brain of female rats plays a neuroprotective role (Nozari et al., 2015a). Acute MK-801 treatment also increases the number of errors during exploration and impaired spatial working memory (Nishiyama et al., 2017). Different processing time of MK-801 has different effects on memory. The earlier the processing time, the greater the adverse effect. This suggests that the disruption of learning and memory by MK-801 results from neurodevelopmental interference, and the early postnatal period is critical for the development of pathways that encode learning and memory (Furuie et al., 2019). The number of arms in the radial maze is variable, some experiments apply the four-arm radial maze (Sampedro-Piquero et al., 2018), and some experiments apply the twelve-arm radial maze (MacQueen and Young, 2020), but the contents tested are similar, which are working memory and reference memory.

PPI is a measure of sensorimotor gating function, which is used to detect deficits in information processing ability and inhibition function. Patients with schizophrenia have impaired sensorimotor gating systems and have PPI deficiency, which is essential for normal cognitive processes. PPI is operated by giving a weak non-startle stimulus 30 to 500 ms before the startle reflex is induced by the auditory source stimulus, and the non-startle stimulus will increase the attenuation amplitude of the startle reflex (Li et al., 2021). After chronic administration of MK-801 to Wistar neonatal rats, PPI defects are observed in both male and female rats on postnatal days 28–30 (Nozari et al., 2015b). Acute MK-801 treatment also induces PPI defects, and PPI is significantly reduced to 81,85 and 90 dB (Prades et al., 2017).

## EE improved the cognitive symptoms of schizophrenia induced by MK-801

EE is a behavioral paradigm that can stimulate cognitive function, and cognitive stimulation in rodents can be achieved by adding new items or rearranging existing items in the cage, thus positively affecting cognitive behavior (Zhu and Grace, 2022). EE can improve the positive, negative, and cognitive symptoms of MK-801 induced schizophrenia. But the improvement of cognitive symptoms is more obvious, which can prevent the spatial learning and memory defects induced by MK-801. And EE is also beneficial to the cognitive function of normal rodents (Nozari et al., 2014). The processing time of EE is flexible, which can be processed in neonatal rodents (Faatehi et al., 2019), in adolescent rodents (Akillioglu et al., 2012), and in adult rodents (Murueta-Goyena et al., 2018). The processing of EE at different stages has a positive effect on the cognitive behavior. The following will review the improvement of EE in MK-801 induced cognitive symptoms in schizophrenia (see Table 1).

In the NOR experiment, EE treatment of Wistar rats can completely reverse the effect of MK-801 on the recognition index of male Wistar rats, but the effect on the novel object exploration ability of female Wistar rats is not obvious (Faatehi et al., 2019). In addition, short-term EE treatment in early adulthood can restore the recognition memory of male Long Evans rats in the MK-801 treatment group, improve cognitive function, and increase the discrimination index. Moreover, EE treatment also has a benign effect on normal Long Evans rats and can increase the discrimination index of control Long Evans rats (Murueta-Goyena et al., 2018).

In the Morris water maze, EE treatment groups show normal spatial learning ability, and the swimming distance and time taken to reach the positioning platform are significantly reduced, indicating that the memory of Long Evans rats is improved (Murueta-Goyena et al., 2019). In addition, EE also enhances the completion efficiency of the Morris water maze task in the control groups, indicating that EE can also increase the cognitive function of normal Wistar rats (Nozari et al., 2014). However, some experiments find that EE treatment after 10 days of MK-801 administration does not reverse the abnormal behavior in the Morris water maze, possibly because MK-801 has caused irreversible negative effects at this time (Akillioglu et al., 2012).

In the Object-in-place experiment, early EE processing will cause Long Evans rats to spend more time exploring the moving object, and the discrimination index is significantly improved, indicating that EE can restore the memory deficit (Murueta-Goyena et al., 2019).

In the PA test, EE treatment can improve long-term memory and increase the latency in the MK-801 treatment groups to enter the dark room. At the same time, it also has a positive effect on normal Wistar rats and can increase the latency in the control groups to enter the dark room (Rahati et al., 2016).

In the eight-arm radial maze, EE can improve the cognitive impairment symptoms and improve the correct rate of completing the maze task (Cortese et al., 2018). However, there are few studies on EE in MK-801 induced learning and memory impairment in rodents. At present, it is only found that EE can' t improve the symptoms of working memory defects in female Wistar rats, which may be related to the characteristics of the experiment itself on the one hand, and the gender of the rats on the other hand (Nozari et al., 2015a).

In PPI experiments, EE can improve PPI deficiency in male Wistar rats, but not in female Wistar rats, and prolonging EE processing time does not affect PPI improvement. The difference in EE improving PPI may be due to the effect of estrogen in female Wistar rats, which may enhance neuronal discharge and affect PPI regulation (Nozari et al., 2015b).

In the above experiments, the improvement of cognitive symptoms of male and female rodents with EE is different, and this gender difference may be related to the difference in sex hormones. Female animals have more estrogen secretion, and estrogen can affect the cognitive behavior and neuronal placement. Moreover, the metabolism of MK-801 in female rodents is slow, and the concentration of MK-801 in female is higher than that in male, so MK-801 has a great influence on female rodents and their behaviors are not easy to improve (Faatehi et al., 2019).

EE can improve the cognitive symptoms of MK-801 induced schizophrenia, including alleviating the learning, memory, and sensory-motor gating dysfunction in the NOR test, Morris water maze, Object-in-place test, PA test, and PPI test in rodent models. However, late EE treatment can't improve the spatial learning ability in the Morris water maze, and EE does not improve the working memory deficit in the eight-arm radial maze test, and the effect of EE on cognitive symptoms in female and male rodents is inconsistent, indicating that EE does not improve the cognitive impairment comprehensively.

## The mechanism of EE in improving cognitive symptoms

Noncompetitive inhibition of the NMDA receptor by MK-801 can induce cognitive symptoms of schizophrenia, but NMDA receptors are widely distributed. As a second messenger, Ca^2+^ caused by NMDA receptor activation participates in many signaling pathways, and the mechanism of action is complex. At present, the specific mechanism of action of MK-801 in inducing cognitive symptoms in schizophrenia is not fully understood. It is also true for EE improving the schizophrenia-like cognitive symptoms induced by MK-801 in rodents. It is generally believed that EE exposes them to more sensory stimuli, which increase the expression of neurotrophic factors, and promote cell proliferation, cell survival, and dendritic maturation, thereby enhancing cognitive function (Ramírez-Rodríguez et al., 2021).

**Figure 1:**
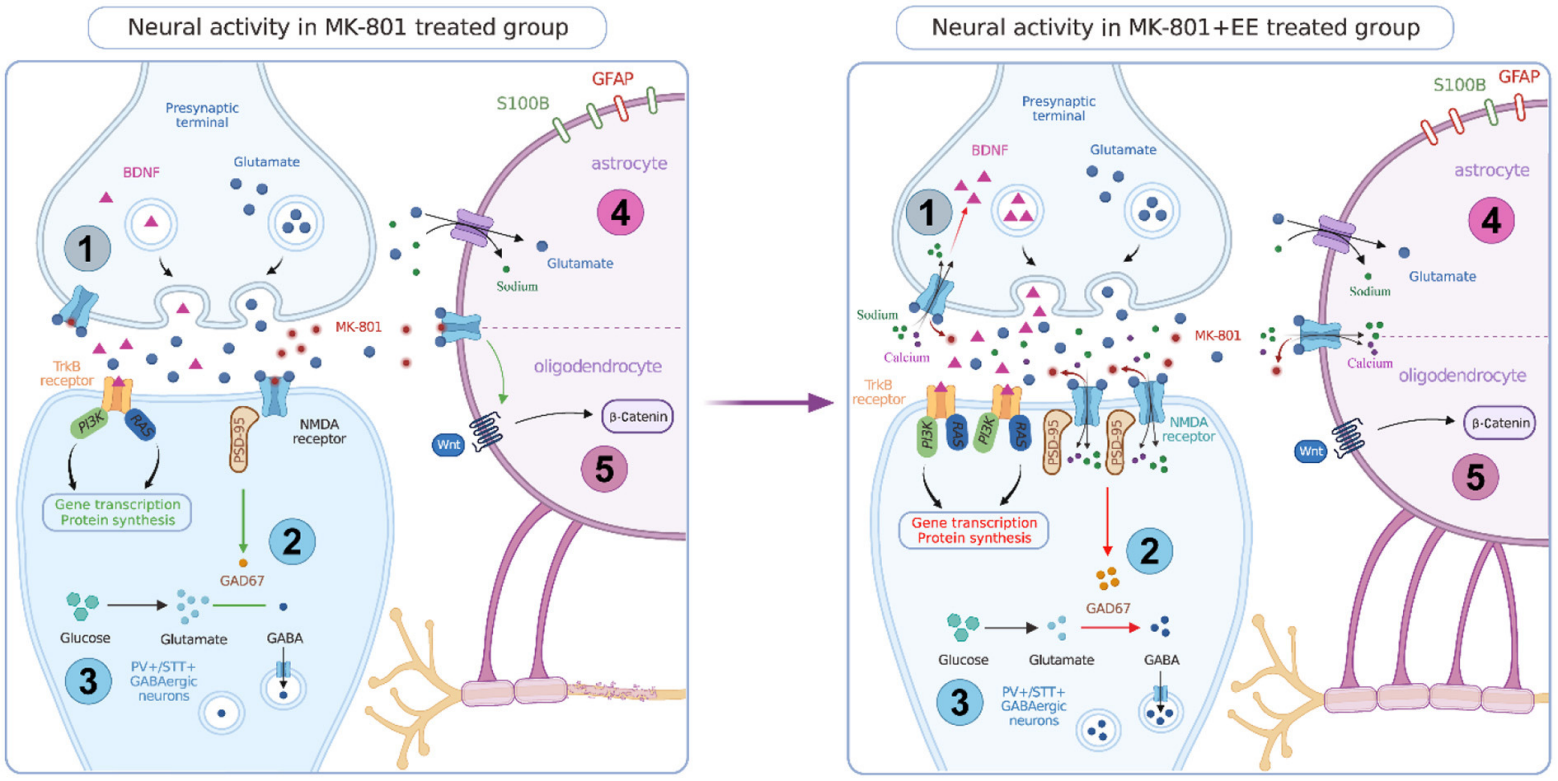
Neural activity in MK-801 and MK-801+EE treated group. EE can promote the expression of BDNF and improve the signaling function of BDNF-TrkB. EE can rescue the blocking effect of MK-801 on NMDAR in interneurons, promote the expression of NMDAR subunits, PSD-95, and GAD 67, and promote GABA neurotransmitter synthesis. EE has a neurorepair effect, which can increase the number of PV+ and SST+ GABAergic neurons and increase the output of inhibitory signals. EE can rescues the regulation of glutamate by astrocytes, increase the number of GFAP positive cells and decrease the number of S100B positive cells. EE can activate Wnt/β signaling pathway in oligodendrocytes and promote myelin synthesis.

In the MK-801 induced cognitive impairment rodent models of NMDA receptor dysfunction, EE affects the expression of neurons, glial cells, and proteins, and improves the cognitive symptoms in a variety of ways. The following will review the possible mechanisms (Figure 1).

### The effect of EE on neurons

Neurons are the main component of the nervous system and a core carrier to exert neuroregulatory function. EE regulates a variety of neurons, increases neuronal plasticity, and promotes neuronal development, to improve the cognitive symptoms of schizophrenia (Ratajczak et al., 2015). MK-801 affects cortical function by reducing the activity of inhibitory interneurons, thereby affecting cognitive function. EE can block the effect of MK-801 on interneurons.

GABA is the main inhibitory neurotransmitter in the central nervous system, which is involved in the excitation/inhibition balance of neuronal activity (Sears and Hewett, 2021). Studies have shown that the expression level of GABA membrane transporters, the density of GABAergic interneurons, and the concentration of GABA in cerebrospinal fluid are reduced in patients with schizophrenia (Marques et al., 2021). MK-801 will preferentially target and block the NMDA receptors on GABAergic parvalbumin (PV) positive interneurons, leading to the decrease of GABAergic neuron activity, reducing the inhibitory effect on pyramidal neurons, and then leading to the increase of cortical excitability (Tarrés-Gatius et al., 2022). The dysfunction of the GABAergic system may be the etiology of cognitive symptoms in schizophrenia. Blocking NMDA receptor in the neonatal period can affect the GABAergic system through multiple mechanisms. MK-801 can reduce the number of PV+ GABAergic interneurons in the hippocampus and PFC, and EE treatment can partially restore the number of these neurons, increasing the output of inhibitory signals (Murueta-Goyena et al., 2018). Somatostatin-positive (SST+) interneurons are also involved in the transmission of inhibitory signals. Their inhibitory effect can regulate sensory and cognitive functions. EE has a nerve repair effect, which can restore the number of SST+ interneurons in the mPFC and hippocampus of mice and improve cognitive function. However, EE does not affect the number of SST+ interneurons in control groups (Murueta-Goyena et al., 2020).

Axon initial segment (AIS) is the initiation site of action potentials and plays a major role in cell excitability. MK-801 can shorten the AIS in the prefrontal cortex, and the mechanism may be that MK-801 causes the inhibition of inhibitory interneurons on the cortex to weaken. The vertebral neurons in the prefrontal cortex are excited, and the AIS is compensatory shortened. EE treatment can increase the plasticity of AIS in the frontal cortex and also prevent MK-801 induced shortening of AIS (Nozari et al., 2017).

### The effect of EE on glial cells

Schizophrenia may involve changes in a variety of glial cells in the brain. Oligodendrocytes are myelinating cells of the central nervous system, myelination in the prefrontal cortex is the basis of normal cognitive function, and myelination depends on the activation of NMDA receptor on oligodendrocytes (Dietz et al., 2020). MK-801 blocks NMDA receptor and inhibits oligodendrocyte activation, thereby reducing myelination in the brain (Lundgaard et al., 2013). EE affects oligodendrocytes and promotes myelination by activating the Wnt/β-catein signaling pathway, increasing Akt and MBP expression and other pathways (Gao et al., 2022).

Astrocytes can synthesize and recycle neurotransmitters, secrete neuromodulators, and participate in the synaptic transmission process. The cell density and morphology of astrocytes in schizophrenia patients are changed, and the expression of cell markers is dysregulated (Notter, 2021). Astrocytes are involved in the metabolism of glutamate in the central nervous system, which can maintain glutamate homeostasis through the uptake and release of glutamate, maintain normal neuronal function and prevent glutamate excitotoxicity (Mahmoud et al., 2019). Pathological changes of astrocytes in the prefrontal cortex are related to cognitive impairment in schizophrenia. GFAP and S100B are two astrocyte markers. MK-801 can interfere with the function of normal astrocytes, reducing the expression of GFAP and increasing the expression of S100B (Rahati et al., 2016). But some studies have found that repeated high doses of MK-801 increased GFAP expression in the hippocampus, which suggested astrocytes were activated by MK-801. It may increase the synthesis of BDNF in the astrocytes. This is a potential action of astrocyte compensatory effect induced by MK-801 (Yu et al., 2015). Early EE corrected MK-801 induced cognitive and behavioral deficits, significantly increased the number of GFAP-positive cells in the prefrontal cortex, and significantly reduced the number of S100B-positive cells (Rahati et al., 2016). Alternatively, EE can also increase the number of GFAP-positive astrocytes in the hippocampus (Zhang et al., 2021).

### Effect of EE on neurotrophic factors

Brain-derived neurotrophic factor (BDNF) is a protein synthesized and secreted by neurons and involved in the growth and differentiation of neurons. The abnormality of BDNF is closely related to schizophrenia and plays a key role in learning and memory. The levels of BDNF in blood, cerebrospinal fluid, and different brain regions of schizophrenia patients are low (Harb et al., 2021). Tyrosine kinase B (TrkB) is a high-affinity receptor for BDNF. Binding of BDNF and TrkB affects neuronal function through the signaling cascade of target tissues (Mehrotra et al., 2022). Studies have shown that MK-801 can not only reduce the expression level of BDNF in prefrontal cortex and hippocampus (Yu et al., 2019), but also reduce the expression of TrkB and affect BDNF-TRKB signaling (Guo et al., 2020). EE treatment can increase the expression of BDNF and improve the signaling function of BDNF-TrkB (Murueta-Goyena et al., 2020). However, early EE treatment can improve the MK-801 induced reduction of BDNF in the hippocampus of male rodents, but it can't improve the reduction of BDNF in female, which may be the reason why EE does not significantly improve NOR in female rodents. In addition, EE also increases the expression level of BNDF in the control groups (Faatehi et al., 2019). In addition to BDNF, EE can also promote the expression of various neurotrophic factors, including nerve growth factor (Zhang et al., 2018), vascular endothelial growth factor (Rostami et al., 2021), and glial derived neurotrophic factor (Takuma et al., 2014), and improve cognitive symptoms in mice.

### Effect of EE on other proteins expression

In addition to BDNF, EE also has an effect on the expression of other proteins. EE can promote the expression of NMDA receptor in the hippocampus of neonatal rodents treated with MK-801. NMDA receptor in the central nervous system mainly consist of tetramers of GluN1 and GluN2A/GluN2B subunits (Benske et al., 2022). MK-801 can down-regulate the expression of the NR1 subunit of the NMDA receptor in the hippocampus, and EE treatment can restore the expression level of NR1. In addition, EE can promote the expression of NR2A and NR2B subunits of the NMDA receptor (Murueta-Goyena et al., 2019). Specific inactivation of the NR1 subunit gene of the NMDA receptor in the CA1 region of the hippocampus leads to memory impairment, and mouse with overexpression of the NR2B subunit show better learning and memory abilities (Grilli et al., 2009). PSD-95 is a postsynaptic protein that regulates the expression of the NMDA receptor. EE treatment can rescue the decrease in PSD-95 expression induced by MK-801, indicating that EE will promote the neurotransmission effect of the NMDA receptor by affecting protein expression (Murueta-Goyena et al., 2019).

MK-801 can also significantly down-regulate a variety of GABA-related proteins including GAD65 (encoding glutamate decarboxylase 65), SYNPR (encoding synaptoporin), GAT3 (encoding sodium and chloride dependent GABA transporter 3), SN1 (encoding sodium coupled neutral amino acid transporter 3), DBI (encoding acyl-CoA binding protein), and CPT1A (encoding carnitine O-palmitoyltransferase 1), which are involved in the process of GABA synthesis, release, reuptake, and recruitment to maintain GABA function and homeostasis (Wang et al., 2021). Among them, glutamic acid decarboxylase 67 (GAD 67) is a GABA synthase. MK-801 can reduce the expression of GAD 67 in the hippocampus, leading to abnormal GABAergic system function, and EE can increase the expression of GAD 67 and promote the recovery of the GABAergic system (Murueta-Goyena et al., 2018).

## Conclusion

Cognitive symptoms are one of the core symptoms of schizophrenia, but there is no effective treatment in clinical practice. The research on cognitive symptoms of schizophrenia is a hot topic nowadays. EE is an environmental intervention and has a significant improvement effect on cognitive symptoms. EE can alleviate a series of learning and memory deficits induced by MK-801, including NOR and Morris water maze. In this review, we summarize the recent literature on MK-801 induced cognitive impairment in rodents, the ameliorating effects of EE on cognitive impairment, and the possible mechanisms involved. After years of research, although people have a certain understanding of the mechanism by which MK-801 induces cognitive symptoms in schizophrenia and EE improves cognitive impairment, there are still some contradictions and problems to be solved in research. For example, in NOR, it is suggested that MK-801 administration above the threshold of 0.5 mg/kg can successfully induce working memory deficits, but some experiments can successfully induce working memory deficits by 0.1 mg/kg MK-801. In the PA test, the damage to long-term memory and short-term memory is different with different concentrations and ways of MK-801 administration. Rodents of different genders have different responses to MK-801 and EE, which may be related to the secretion of estrogen and the metabolism of MK-801, but the specific mechanism remains to be studied. Alternatively, although EE improves experimentally in response to abnormal cognitive behavior, it is still not possible to determine to what extent it plays a role during treatment and which components of EE are more effective in treatment. As an environmental intervention, EE can't completely control the symptoms of cognitive impairment in schizophrenia, and it is currently only used as a preventive method. Therefore, the research on cognitive symptoms of schizophrenia still has a long way to go. It is believed that with in-depth research, new pathological mechanisms will continue to emerge, and new treatment methods will be more perfect to prevent and control the occurrence and development of the disease, to bring good news to patients suffering from schizophrenia.

## Author contributions

WW designed and supervised the project. JX wrote the original draft. All authors reviewed and edited the text and approved the submitted version.

## Funding

This study was supported by the Natural Science Foundation of China (82071536 to WW), Shaanxi Provincial Key Research and Development Program (2020ZDLSF01–09 to SW), and the CAS Key Laboratory of Brain Connectome and Manipulation (2019DP173024 to WW).

## Conflict of interest

The authors declare that the research was conducted in the absence of any commercial or financial relationships that could be construed as a potential conflict of interest.

## Publisher's note

All claims expressed in this article are solely those of the authors and do not necessarily represent those of their affiliated organizations, or those of the publisher, the editors and the reviewers. Any product that may be evaluated in this article, or claim that may be made by its manufacturer, is not guaranteed or endorsed by the publisher.
